# High-resolution ion mobility based on traveling wave structures for lossless ion manipulation resolves hidden lipid features

**DOI:** 10.1007/s00216-024-05385-8

**Published:** 2024-06-27

**Authors:** Allison R. Reardon, Jody C. May, Katrina L. Leaptrot, John A. McLean

**Affiliations:** Center for Innovative Technology, Department of Chemistry, Vanderbilt Institute of Chemical Biology, Vanderbilt Institute for Integrative Biosystems Research and Education, Vanderbilt-Ingram Cancer Center, Vanderbilt University, Nashville, TN 37235 USA

**Keywords:** Lipidomics, Ion mobility-mass spectrometry, High-resolution ion mobility-mass spectrometry, Lipid structure, Structures for lossless ion manipulation

## Abstract

High-resolution ion mobility (resolving power > 200) coupled with mass spectrometry (MS) is a powerful analytical tool for resolving isobars and isomers in complex samples. High-resolution ion mobility is capable of discerning additional structurally distinct features, which are not observed with conventional resolving power ion mobility (IM, resolving power ~ 50) techniques such as traveling wave IM and drift tube ion mobility (DTIM). DTIM in particular is considered to be the “gold standard” IM technique since collision cross section (CCS) values are directly obtained through a first-principles relationship, whereas traveling wave IM techniques require an additional calibration strategy to determine accurate CCS values. In this study, we aim to evaluate the separation capabilities of a traveling wave ion mobility structures for lossless ion manipulation platform integrated with mass spectrometry analysis (SLIM IM-MS) for both lipid isomer standards and complex lipid samples. A cross-platform investigation of seven subclass-specific lipid extracts examined by both DTIM-MS and SLIM IM-MS showed additional features were observed for all lipid extracts when examined under high resolving power IM conditions, with the number of CCS-aligned features that resolve into additional peaks from DTIM-MS to SLIM IM-MS analysis varying between 5 and 50%, depending on the specific lipid sub-class investigated. Lipid CCS values are obtained from SLIM IM (^TW(SLIM)^CCS) through a two-step calibration procedure to align these measurements to within 2% average bias to reference values obtained via DTIM (^DT^CCS). A total of 225 lipid features from seven lipid extracts are subsequently identified in the high resolving power IM analysis by a combination of accurate mass-to-charge, CCS, retention time, and linear mobility-mass correlations to curate a high-resolution IM lipid structural atlas. These results emphasize the high isomeric complexity present in lipidomic samples and underscore the need for multiple analytical stages of separation operated at high resolution.

**Graphical abstract:**

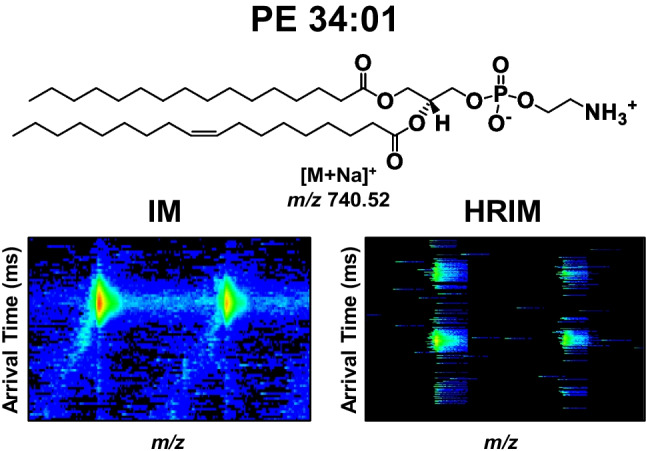

**Supplementary Information:**

The online version contains supplementary material available at 10.1007/s00216-024-05385-8.

## Introduction

Mass spectrometry (MS) is one of the premier analytical techniques for molecular characterization. MS represents several different technologies that discriminate ions based on their intrinsic mass, some of which are considered high-resolution MS (HRMS, resolution > 10,000), which allows for finer mass measurements to be achieved with greater precision. However, isomers possess identical chemical formulae and thus are not able to be resolved with HRMS alone. Operating multiple MS stages in tandem (MS/MS) allows for mass-resolved ion dissociation, which provides additional structural information that in some cases allows for isomeric differentiation, although many isomers have similar fragmentation spectra and thus are indistinguishable with MS/MS.

Lipids are biomolecules that significantly contribute to the structure and function of living organisms. They are known for a prevalence of isomers, which primarily result from variability in their hydrophobic regions. Specificity in the identification of lipid isomers is important, because of their potential as diagnostic biomarkers for disease states and can in some cases lessen the potential severity of a specific disorder [1]. For example, cardiovascular disease (CVD) risk has been attributed to four specific ceramides: d18:1/16:0, d18:1/18:0, d18:1/24:0, and d18:1/24:1, which are isomeric to numerous other lipids with variations in acyl tail length and double bond location [2]. While these individual lipids can serve as biomarkers, in many instances groups of similar lipids can also signal the potential onset of a disorder [2]. Thus, the ability to isolate entire classes of lipids and their isomeric components has the potential to benefit diagnoses in clinical chemistry and promote better patient treatment strategies.

According to a 2015 survey of the molecular coverage of the world’s largest chemical repository, PubChem, there are over 60 million unique compounds that are known to exist within 10 to 1,000 Da. Focusing in on a narrow 1 ppm window (354.165 ± 0.0015 Da), there are estimated to be over 11,000 existing structures, underscoring the importance of high chemical resolution measurements [3]. An analytical separation technique now routinely integrated with MS is ion mobility (IM-MS). The combination of IM with MS results in a high throughput separation that is structurally selective to size/shape, specifically charge-to-collision cross section (*z/Ω* by IM) and mass-to-charge (*m/z* by MS) [4]. The IM dimension also provides a reproducible analytical measurement, the collision cross section (CCS), which is useful for compound characterization and alignment of measurements across different laboratories and techniques. Among the different IM separation approaches, drift tube IM (DTIM) can provide direct measurement of CCS values while accessing modest IM resolutions (single peak resolving power, R_p_ ~ 50) [5, 6]. Recent developments in IM have targeted increases in the resolving power of the separation, which have resulted in the development of high-resolution ion mobility (HRIM) techniques such as cyclic IM (cIM), trapped IM spectrometry (TIMS), and the post processing technique HR demultiplexing (HRdm), all of which can access R_p_ values in excess of 200 [7, 8]. A particularly promising technology based on traveling wave structures for lossless ion manipulation (SLIM IM) can achieve high resolving powers (R_p_ > 200) for all species across the IM range used for analysis, enabling full HRIM-MS spectra to be acquired within the timescale of chromatographic separations, while demonstrating high selectivity in resolving various isomer systems [9–12]. This is in contrast to IM strategies that provide high resolving powers for a narrow range of ion mobilities that are selected for high resolving power separation.

A theoretical framework to understand what types of isomers could be resolved by IM with increasing levels of resolving power was proposed by Dodds et al*.* [13]. Intuitively, as the percent difference in CCS for a pair of isomers becomes smaller (more structurally similar), higher resolving powers are needed for separation. For example, in order to achieve 50% separation (half height resolved), constitutional isomers were estimated to require a resolving power of ~ 50, whereas cis/trans isomers required a resolving power of approximately 200, and diastereomers exhibited very similar CCS values, necessitating a resolving power of at least 300 for partial resolution [13]. Lastly, enantiomers (“mirror-image” isomers) were indistinguishable using the conventional resolution capability of the DTIM used in the study, and thus were estimated to need very high IM resolving powers to begin to assess their separation, and these systems may not even exhibit structural differences that are resolvable by IM. The high R_p_ accessed by SLIM IM-MS across a broad range of mass enables complex lipid isomer systems to be distinguished from each other to support more specific structural identifications in lipidomic studies. More specifically, the ability to separate cis/trans isomers, double bond positional isomers, and stereoisomers can be achieved with SLIM IM. However, the high resolving power capabilities of SLIM IM-MS to differentiate isomers in complex, biologically-derived samples has not been fully investigated, nor has the potential of CCS alignment for providing high-confidence structural annotations in these multidimensional workflows been assessed [14, 15].

**Fig. 1:**
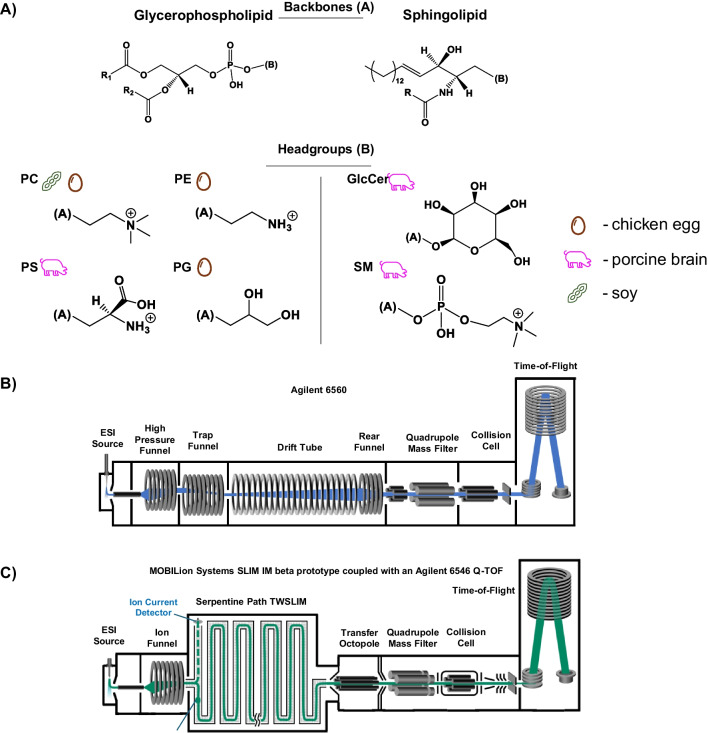
**A** Generalized chemical lipid structures of total lipid extracts studied, including the lipid category backbone and the class headgroups. The source of each lipid extract is indicated with an icon representing egg, pig, or soybean origins. **B** Schematic of the DTIM-MS instrument used for the reference CCS analyses. **C** Schematic of the SLIM IM-MS (beta prototype) used for the HRIM analyses

In this work, we investigate the capabilities of HRIM-MS analysis for providing accurate lipid identification. We begin by analyzing mixtures of several commercially-available isomeric lipid standards incorporating multiple isomeric types, to assess the ability to resolve challenging systems and to provide benchmarks as to what specific lipid isomer systems can be resolved by HRIM. We then focus on analyzing more complex lipid samples in the form of total lipid extracts, each consisting primarily of lipids of a specific structural sub-classification and thus, putatively similar structures. Within each lipid extract, we observe features previously reported as lipid species comprised of a single DTIM peak [16], which are resolved into additional SLIM IM peaks under the higher resolving powers achieved via SLIM IM-MS. These additional features observed under HRIM conditions were then tentatively identified using additional pieces of analytical information (outlined below) and supported by CCS-based mathematical fits to the observed mobility-mass correlations generated for each lipid subclass. A total of 225 calibrated ^TW(SLIM)^CCS values were curated from the HRIM analysis as obtained for seven lipid extracts (Fig. 1A) in positive ion mode.

## Methods

### Materials and solvents

High-purity (Optima grade) solvents including methanol, chloroform, water, acetonitrile, 2-propanol (IPA), and formic acid were purchased from Fisher Scientific (Hampton, NH). Ammonium formate was purchased from Sigma-Aldrich and was used as a mobile phase additive. A tuning mixture containing symmetrically branched hexakis(fluoroalkoxy)-phosphazenes (HFAPs, ESI-L low concentration tuning mixture, Agilent Technologies) was used for calibrating mass-to-charge (*m/z*) and CCS for both instruments in this study. Purified TLC fractions of total lipid extracts were purchased from Avanti Polar Lipids (Birmingham, AL) as lyophilized powders, reconstituted in chloroform, and prepared to a final concentration of 10 µg/mL in 1:2 chloroform:methanol for analysis. Total lipid extracts including glycerophosphocholine (PC, both chicken egg and soy), glycerophosphoethanolamine (PE, chicken egg), glycerophosphoserine (PS, porcine brain), glycerophosphoglycerol (PG, chicken egg), glucosylceramide (GlcCer, porcine brain), and sphingomyelin (SM, porcine brain). Lipid isomer standards were purchased from Cayman Chemical (Ann Arbor, MI) and Avanti Polar Lipids. PC and PE standards were prepared at 10 µg/mL in 1:1 acetonitrile:methanol, triglyceride (TG) standards were prepared at 40 µM equimolar concentrations in 1:1 methanol:IPA with 2 mM ammonium acetate, and diglyceride (DG) standards were prepared at 40 µM equimolar concentrations in 1:1 methanol:IPA.

### Instrumentation

Samples were analyzed via DTIM-MS (6560 IM-Q-TOF, Agilent Technologies) and SLIM IM (both beta prototype and MOBIE platform, MOBILion Systems, Inc.) interfaced with a HRMS (6546 Q-TOF, Agilent). Conceptual instrument diagrams are shown in Fig. 1B and C. The main difference between the two SLIM IM platforms (beta prototype vs MOBIE platform) is the orientation of the SLIM board (horizontal vs vertical, respectively). Samples were introduced via flow injection analysis (FIA) using a liquid chromatography system (1290 Infinity II, Agilent) and were ionized by positive electrospray ionization (ESI) (Jet Stream, Agilent). All platforms used ultra-high purity nitrogen drift gas in the mobility stage, yielding nitrogen-based CCS measurements (CCS_N2_) [17].

### Data acquisition

For both DTIM-MS and SLIM IM-MS platforms, the ESI source used was identical and operated in positive ion mode under the following conditions: nebulizer pressure, 20 psi; sheath gas flow rate, 12 L/min; sheath gas temperature, 275 °C; drying gas flow rate, 5 L/min; drying gas temperature, 325 °C; capillary voltage, 4000 V; entrance nozzle voltage, 2000 V [9].

For DTIM-MS acquisition, total lipid extracts were infused using a 3-min FIA method [18] with an injection volume of 10 µL and a carrier solvent of 0.1% formic acid in 1:1 methanol:water at a flow rate of 70 µL/min. The drift tube was operated under 3.95 Torr nitrogen gas, along with the following additional parameters: ion trap fill time, 20 ms; ion trap release time, 300 µs; drift tube entrance, 1474 V; drift tube exit, 224 V; rear funnel entrance, 217.5 V; rear funnel RF, 150 V_pp_; rear funnel exit, 45 V; and IM hexapole entrance, 41 V [19]. The Q-TOF was operated in the low mass range (*m/z* 50–1700), the ion slicer was operated at high sensitivity, and the digitizer operated at 2 GHz extended dynamic range. Single-field ^DT^CCS measurements were obtained using HFAP drift times calibrated to reference ^DT^CCS values [19].

For SLIM IM-MS acquisition, total lipid extracts were first separated using reversed-phase liquid chromatography (RPLC) with a 1290 Infinity II LC system (Agilent). Mobile phases consisted of both 10 mM ammonium formate and 0.1% formic acid in (A) H_2_O and (B) 60:36:4 IPA:ACN:H_2_O. RPLC was performed using a C-18 column (HypersilGold 1.9 µm, 2.1 mm × 100 mm, Thermo Fisher) at 40 °C with a flow rate of 250 µL/min over a 30-min gradient (Figure S1) [20]. The SLIM IM chamber was operated at 2.50 Torr. TW-based separation was performed using a wave speed of 180 m/s and a peak-to-peak wave amplitude of 40 V_pp_, optimized for IM resolution based on work from May et al*.* [9]. Specific SLIM IM method parameters were chosen to be optimal for the mass range of lipids observed. Data were acquired using MassHunter Acquisition (v. 9.0, Agilent) and EyeOn software (v. 0.3.1.0, MOBILion).

### Lipid identification and nomenclature

Lipids were identified by database matching of *m/z* (± 5 ppm) to the Unified CCS Compendium from Picache et al*.* [21, 22] and LIPID MAPS [23, 24]. The LIPID MAPS nomenclature is used throughout the manuscript. Lipids are annotated by their subclass followed by the total number of carbons and the degree of unsaturation of the acyl tails. For example, PC 36:01 denotes the PC subclass with 36 total tail carbons and one site of unsaturation. A lipid feature refers to signal observed at a unique *m/z* and CCS, which are extracted from a narrow RT (or FIA) range.

### Data processing

Lipid IM-MS features were manually extracted as peak centroids using MassHunter IM-MS Browser (v. 10.0.1, Agilent). ^TW(SLIM)^CCS values were calibrated via a 3rd order polynomial using the HFAP ions, and a subclass-specific linear correction factor (based on the average bias of a group of non-splitting lipid features in HRIM) was utilized to align calibrated ^TW(SLIM)^CCS values to within 2% average bias to reference ^DT^CCS values (PG correction factor shown in Figure S2) as described by Rose et al*.* [20, 25]. Note that correction factors must be reevaluated for any changes in instrumental settings (*i.e*. for different laboratories). In this work, the correction factors obtained for each subclass were directly multiplied to the initial ^TW(SLIM)^CCS resulting from the 3rd order polynomial to get the final calibrated ^TW(SLIM)^CCS value (reported in Table S1). CCS calibration, biases, and SLIM IM correction factors were calculated in Excel (Microsoft).

## Results and discussion

### HRIM separation of isomeric lipid standard mixes

**Fig. 2:**
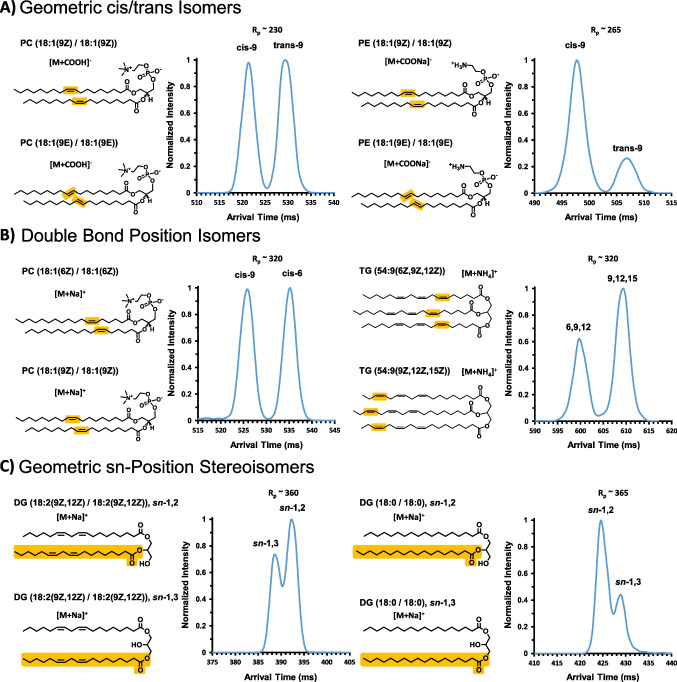
HRIM analysis of lipid isomer standard mixtures. **A** Separation of PC and PE geometric cis/trans isomers. **B** Separation of PC and TG double bond positional isomers. **C** Separation of DG geometric sn-positional stereoisomers. Peak assignments were confirmed by analysis of the individual isomers

Previously, May et al*.* [9] evaluated selected lipid isomer systems via SLIM IM-MS. In this study, we focus on direct resolution of mixed lipid standards, which represent three different forms of lipid isomers. In each of these examples, the highest abundance ion form is presented, namely [M+COOH]^−^, [M+Na]^+^, and [M+Na]^+^, in Fig. 2A–C, respectively. Figure 2A shows the HRIM analysis of geometric cis/trans isomers. For these two PC and PE lipid isomer standard mixtures, the only difference within each subclass is the geometry of the double bond found on each fatty acid tail, as highlighted on their respective structures in Fig. 2A. The PC isomers, PC (18:1(9Z) / 18:1(9Z)) and PC (18:1(9E) / 18:1(9E)), display near-baseline separation, whereas the PE isomers, PE (18:1(9Z) / 18:1(9Z)) and PE (18:1(9E) / 18:1(9E)), show a complete baseline separation. For both cases, the cis-configuration adopts a more compact gas-phase structure than the trans-configuration, as indicated by the faster arrival time of the cis-configuration, and these results are in agreement with the analyses of the PC cis/trans isomers by both Kyle et al*.* and Jeanne Dit Fouque et al*.* [15, 26]. Figure 2B shows the HRIM arrival time distributions for double bond positional isomers including a mixture of PC (18:1(6Z) / 18:1(6Z)) with PC (18:1(9Z) / 18:1(9Z)) and a mixture of TG (54:9(6Z,9Z,12Z)) with TG (54:9(9Z,12Z,15Z)). Both sets of double bond positional isomers display near baseline resolution when analyzed by HRIM. The PC cis-9 peak had a shorter arrival time than the PC cis-6 peak, indicating a more compact structure when the site of unsaturation is located closer to the end of the acyl tail. This finding supports that of Kyle et al*.* who reported fatty acids had smaller conformations when the site of unsaturation was located further from the lipid headgroup [15]. For the triglycerides, we observe TG (54:9(6Z,9Z,12Z)) at an earlier arrival time, and thus a more compact structure, than TG (54:9(9Z,12Z,15Z)). Considering just the positions of the sites of unsaturation, the TG isomer results are the opposite of the PC isomers just described. This suggests that when more than one site of unsaturation is present on an acyl tail, or an additional degree of structural freedom is incorporated (i.e. a third acyl tail), a combination of the headgroup and third acyl tail is able to interact with those sites of unsaturation to yield a more compact structure. However, the third acyl tail likely contributes to the gas-phase compaction to a lesser degree than the headgroup. In Fig. 2C, we evaluate the separation of geometric *sn*-position stereoisomers including two sets of diglycerides: DG (18:2(9Z,12Z) / 18:2(9Z,12Z)), *sn*-1,2 with DG (18:2(9Z,12Z) / 18:2(9Z,12Z)), *sn*-1,3, and DG (18:0 / 18:0), *sn*-1,2 with DG (18:0 / 18:0), *sn*-1,3. Though the single peak resolving power achieved in these two diglyceride separations was higher (R_p_ ~ 360–365) than those achieved with the previous four isomer mixtures (R_p_ ~ 230–320), the differences in arrival times were relatively smaller, resulting in approximately half-height separation between both sets of diglyceride peaks. This indicates that the structural difference in geometric *sn*-position stereoisomers has only a minor impact on their gas-phase conformations, requiring higher resolving power to separate them. For the mixture of the DG 18:2/18:2 isomers, the *sn*-1,3 isomer appears before the *sn*-1,2 isomer, suggesting that with multiple double bonds on the acyl tail, the further apart the acyl tails are to each other, the more compact structure the isomer can adopt in the gas phase. However, the opposite arrival time ordering is observed when comparing the saturated DG 18:0/18:0 isomers. Specifically the *sn*-1,2 isomer appears before the *sn*-1,3 isomer, suggesting that the absence of double bonds in the acyl tails changes the gas-phase conformations adopted by the diglycerides. The arrival time orderings that are observed experimentally for these lipid isomers are nonetheless challenging to predict from an inspection of the primary structure alone, which underscores the importance of empirical data such as these for interpreting results as well as training prediction algorithms and theoretical gas-phase mobility behavior. Jeanne Dit Fouque et al*.* also analyzed both the DG (18:2(9Z,12Z) / 18:2(9Z,12Z)), sn-1,2 and DG (18:2(9Z,12Z) / 18:2(9Z,12Z)), sn-1,3 geometric *sn*-position stereoisomers using TIMS-based HRIM and observed a single IM profile for their corresponding mixtures, whereas our results show two resolved IM features for these DG stereoisomers due to the higher resolving powers achieved with SLIM-based HRIM (R_p_ ~ 360 for SLIM IM compared to R_p_ ~ 230 for TIMS) [26]. In summary, the geometric cis/trans and the double bond positional isomers achieved near or full baseline separation with HRIM via SLIM IM-MS, whereas the geometric *sn*-positional stereoisomers exhibited approximately half-height peak-to-peak separation. These results are in general agreement with previously estimated IM resolving powers needed to separate differing levels of isomeric complexity, which was initially inferred from amino acid isomers [13].

### Evaluating separation power of HRIM vs. DTIM

DTIM is generally able to access resolving powers of ~ 50, which can lead to multiple lipid isomers observed as a single unresolved peak [6]. The further resolution of these peaks into multiple features can be achieved with HRIM via SLIM IM-MS (R_p_ > 200), [9]. To assess the extent of isomeric complexity present in biologically-derived lipid mixtures, seven total sub-class specific lipid extracts were evaluated from the glycerophospholipids (PE, PC egg, PC soy, PS, and PG) and sphingolipids (GlcCer and SM) classes. As shown in Fig. 1A, glycerophospholipids contain two acyl tails varying in chain length and number of double bonds whereas sphingolipids have a fixed sphingosine backbone and one acyl tail varying in chain length and number of double bonds. Lipid annotations with IM are generally limited to the total numbers of acyl tail carbons and sites of unsaturation. While not generally reported from MS or IM-MS data, additional specificity in lipid characterization such as double bond locations and stereochemistry can be obtained by integrating other techniques, such as ozonolysis and fragmentation techniques with IM-MS [27, 28].

**Fig. 3:**
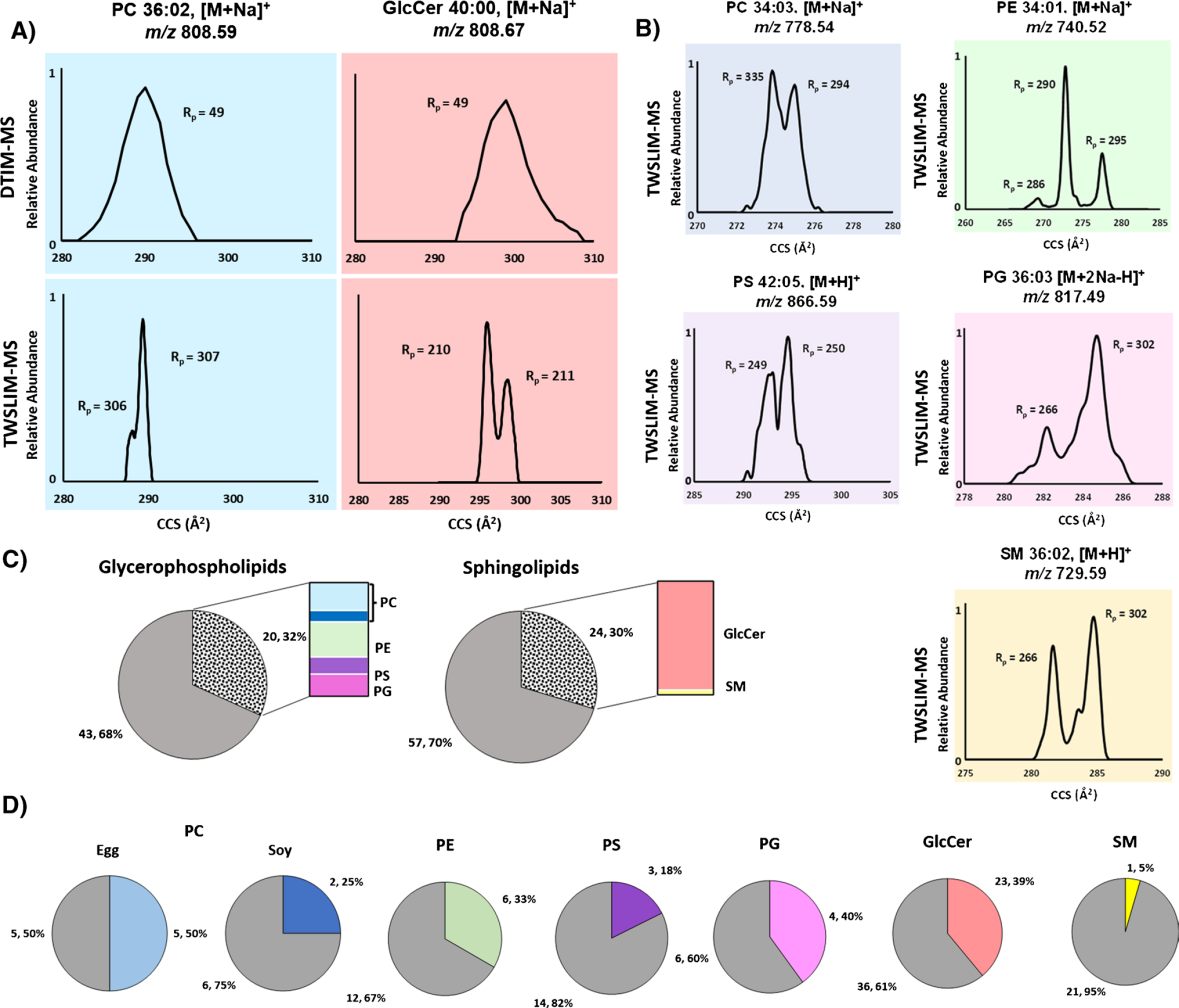
Summary of the IM analysis using both conventional resolution IM (DTIM) and HRIM (SLIM IM) MS. **A** PC and GlcCer separations in CCS space. **B** Examples of lipid IM profiles exhibiting multiple features with HRIM. **C** Lipid features observed with DTIM-MS (gray) compared to SLIM IM-MS (speckled pattern) per lipid category. Color region shows a comparison of the number of splitting features for each total lipid extract. **D** Lipid features observed with DTIM-MS (gray) and SLIM IM-MS (color) per lipid subclass. The region of color indicates the number of lipid features that resolve into multiple peaks with HRIM that were observed as a single feature with DTIM-MS

Analyses via DTIM-MS yielded a total of 144 identified lipid features, (63 glycerophospholipids and 81 sphingolipids), whereas SLIM IM-MS analysis yielded a total of 188 lipid features (83 glycerophospholipids and 105 sphingolipids). The higher number of lipid features obtained by HRIM is driven by the high resolving power of the platform, but also due to the enhanced sensitivity afforded by the use of LC vs FIA, the former of which reduces ion suppression effects by limiting the amount of sample introduced to the ion source at any given time. This study utilized FIA to obtain the conventional resolution IM profiles, while the rainder of the analyses with SLIM IM-MS utilized LC for sample introduction. It is important to highlight that only lipid features observed with DTIM-MS were investigated with SLIM IM-MS and thus the Fig. 3 summaries preclude features uniquely observed in HRIM analysis. Figure 3A provides a direct comparison of DTIM-MS and SLIM IM-MS lipid features projected in CCS space to highlight how a single feature detected with DTIM-MS can yield multiple peak features with SLIM IM-MS. These lipid features from PC egg and GlcCer lipid extracts were partially resolved into two features with SLIM IM-MS. Figure 3B expands the SLIM IM-MS analysis to the PS, PE, SM, PC soy, and PG lipid extracts where one lipid feature from each subclass resolves into multiple peaks under HRIM. Specifically, the PE 34:01 [M + Na]^+^ lipid feature (green shading) exhibits three IM peaks with SLIM IM compared to a single peak observed with DTIM (Figure S3). Figure 3C highlights the percentage of additional features observed from each lipid class using high resolving power IM (speckled pattern) as a fraction of the original number of features detected with DTIM-MS at conventional resolving power (gray), with a sub-class specific summary provided in Fig. 3D. For example, PC egg displayed 10 DTIM features, but with SLIM IM, five of those 10 features are now shown to be splitting into more than one feature. About 32% of glycerophospholipids displayed features that yielded multiple IM peaks with SLIM IM-MS whereas sphingolipids were found to have about 30% of lipid features resolve into additional IM peaks. Since glycerophospholipids have two varying acyl tails compared to sphingolipids where one of the two acyl tails is a fixed sphingosine backbone, glycerophospholipids have more degrees of freedom and more potential for isomerism. The SM result, with the lowest percentage of splitting lipid features, coincides with this reasoning with only 5% additional HRIM features. An extensive list of each lipid feature observed including numerous new lipid features observed with SLIM IM-MS is contained in Table S1.

**Fig. 4:**
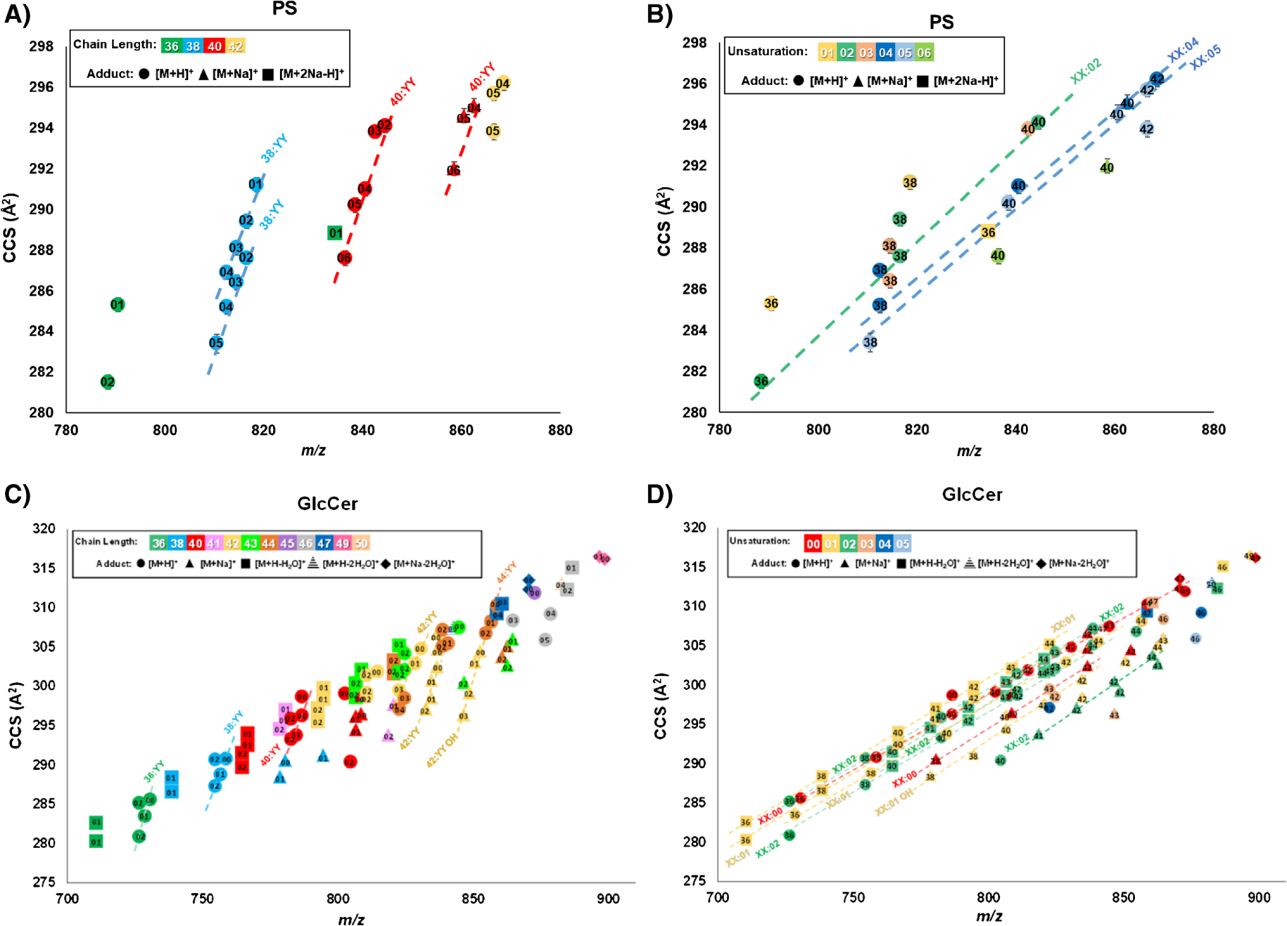
Mobility-mass correlations of PS and GlcCer total lipid extracts. **A** PS chain length linear correlations. **B** PS unsaturation linear correlations. **C** GlcCer chain length linear correlations. **D** GlcCer unsaturation linear correlations. **A**, **C** Chain length trendline identities labeled by total carbons on acyl tails:YY where YY indicates the total number of double bonds, with the number of double bonds annotated with the data points. **B**, **D** Unsaturation trendline identities are labeled by XX:total number of double bonds on the acyl tails where XX indicates the total number, with the number of carbons annotated with the data points. Trendlines each contain *n* ≥ 3 number of lipid features. Adduct type is designated by the shape of the marker

In prior work from Leaptrot et al*.*, linear mobility-mass correlations for these seven lipid sub-classes were mapped in detail using conventional resolution DTIM-MS analysis, which allowed a conformational atlas of lipids to be constructed [16]. In this HRIM study, new mobility-mass correlations are described within the higher resolution separation of lipid features observed with SLIM IM-MS. To quantitatively map these empirical correlations, calibrated ^TW(SLIM)^CCS values were first projected as a function of mass-to-charge. Unambiguous lipid identifications were assigned from a combination of accurate mass, ^DT^CCS, and retention time, and known lipids were identified within each linear mobility-mass correlation to promote secondary annotation of ambiguous lipid features residing along the same trend. Figure 4 shows two types of lipid correlation trendlines where lipids of the same ion form are grouped by either (1) shared acyl tail chain length (Fig. 4A for PS, Fig. 4C for GlcCer) or (2) shared degree of unsaturation (Fig. 4B for PS, Fig. 4D for GlcCer). The PS chain length trendlines in Fig. 4A include two trends for 38:YY [M+H]^+^ of particular interest. These correlations contain three mass-to-charge values that were observed with single ^DT^CCS measurements, that each resolved into two distinct ^TW(SLIM)^CCS values for PS 38:02, PS 38:03, and PS 38:04, though further analysis would be required to discern greater specificity in the identification of these isomeric lipid pairs. The PS unsaturation trends in Fig. 4B include three [M+H]^+^ adduct trendlines, all with similar slope. Additional correlation lines are also observed in the HRIM analysis for GlcCer, including newly-resolved chain length (7) and unsaturation (10) correlations. Mobility-mass correlation plots for the other five total lipid extracts investigated are shown in Figure S5. All lipid extracts exhibited additional measurements that were not fit to linear models due to insufficient numbers of data points (*n* < 3).

From this work, a total of 225 calibrated ^TW(SLIM)^CCS values are reported across the seven total lipid extracts studied and confirmed with at least one additional replicate, with 37 of these ^TW(SLIM)^CCS measurements corresponding to features newly observed with HRIM-MS. Corresponding mobility-mass correlations are shown in Fig. 4. The high number of features uncovered by HRIM analysis underscore the isomeric complexity present in biologically-derived lipids, and the resulting lipid mobility-mass correlations can be utilized in nontargeted discovery workflows to increase confidence in potential lipid identifications.

### SLIM IM-MS intra-platform comparisons

To validate the HRIM observations and assess the reproducibility of the ^TW(SLIM)^CCS calibration methodology, five total lipid extracts were chosen for analysis on a separate, commercial equivalent MOBIE platform. The same samples and method parameters were duplicated for this follow-up analysis. Nearly identical lipid features were observed between the two platforms, despite a difference of several (10) months between acquisitions. Figure S6 compares the HFAP ions obtained with the same method settings on both SLIM IM platform variants. The HFAP ions were found to have higher arrival times on the MOBIE platform than on the beta prototype, which resulted in a slight 0.6% increase (3–4 Å^2^) in ^TW(SLIM)^CCS values, thus updated correction factors were required for the second step of the ^DT^CCS alignment.

Figure S7 shows the percent biases for the PC, PE, GlcCer, and PG lipid subclasses both before and after applying a class-specific linear correction factor for each subclass, which minimizes the overall percent bias for each class of lipids. Before applying this correction, the 3rd order polynomial calibration using HFAP ions brings the lipid ^TW(SLIM)^CCS values obtained from SLIM IM to within ca. 4% of the reference ^DT^CCS values obtained from DTIM. Applying this linear correction lowers the overall percent bias effectively to zero for all lipids selected. Whereas this second step in the calibration was not necessary for the intra-platform comparisons, applying the linear correction factors reported in Table S3 allowed ^TW(SLIM)^CCS values to be properly aligned (unified) to the reference ^DT^CCS values. An average of the correction factors for the four subclasses studied with MOBIE platform is suggested as the linear correction factor for the SM and PS subclasses in future analyses.

## Conclusions

In this study, we evaluated HRIM measurement capabilities of SLIM IM-MS for accurate lipid annotation. SLIM IM analysis of various lipid isomer standards indicated that the geometric cis/trans isomers and the double bond positional isomers can be resolved at baseline resolution, however the geometric sn-positional stereoisomers exhibit only partial resolution. The analysis of seven total lipid extracts spanning the glycerophospholipid and sphingolipid classes revealed numerous additional features with HRIM that were not observed with conventional resolution IM. When comparing the same extracts, the number of single lipid features, which resolved into additional features varied from 5% (SM) to 50% (PC egg) due to the higher resolving powers achieved with SLIM IM-MS. These observations generally correlated to the number of isomers expected for each lipid sub-class, e.g., more features were observed for glycerophospholipids and glycolipids than for sphingolipids. Of the glycerophospholipid and sphingolipid features observed, an average 30% of each lipid class was resolved into an additional feature under HRIM, which is similar to the number of isomers that were previously observed from a large (*n* = 1246) CCS survey of primary human metabolites (31%) [29]. When plotting the resulting ^TW(SLIM)^CCS values by their mass-to-charge, empirical lipid mobility-mass correlations observed in the HRIM data corresponded to the summed number of carbon atoms in the alkyl chains and total sites of unsaturation. These correlations are referenced to *m/z* and standardized ^DT^CCS values and thus can be utilized to aid future lipid identification on a variety of IM-MS platforms. The SLIM IM beta prototype was compared to the MOBIE platform by analyzing five of the same lipid extracts on each platform. Once alignment to DTIM was established, a HRIM database of 225 calibrated lipid ^TW(SLIM)^CCS values was compiled, which corroborated the presence of linear lipid correlations previously observed with DTIM-MS. In instances where high resolving power results in multiple lipid structures for the same species, each resolved feature is annotated with a corresponding CCS value. Thus, a CCS database of HRIM measurements can facilitate the alignment of each resolved feature to a reference CCS, improving the confidence in identifying the corresponding lipid by correlating its assignment to multiple descriptors. These ^TW(SLIM)^CCS measurements and corresponding empirical trends will benefit future untargeted lipidomics analyses on HRIM platforms and will provide a useful resource to support lipid annotation assignments with greater structural specificity.

## Supplementary Information

Below is the link to the electronic supplementary material.

Supplementary file1 (DOCX 883 KB)
