# Supplementary material for: High-resolution ion mobility based on traveling wave structures for lossless ion manipulation resolves hidden lipid features
Source: Anal Bioanal Chem. 2024 Jun 27;416(25):5473–83. doi: 10.1007/s00216-024-05385-8 (PMC11427608; doi:10.1007/s00216-024-05385-8)
Supplement: Supplementary file 1 — Supplementary file1 (DOCX 883 KB) [file 216_2024_5385_MOESM1_ESM.docx]

Supporting Information

High-Resolution Ion Mobility Based on Traveling Wave Structures for Lossless Ion Manipulation Resolves Hidden Lipid Features

Allison R. Reardon, Jody C. May, Katrina L. Leaptrot, and John A. McLean*

Center for Innovative Technology, Department of Chemistry, Vanderbilt Institute of Chemical Biology, Vanderbilt Institute for Integrative Biosystems Research and Education, Vanderbilt-Ingram Cancer Center, Vanderbilt University, Nashville, TN, 37235, United States

* Corresponding Author Email: [john.a.mclean@vanderbilt.edu](mailto:john.a.mclean@vanderbilt.edu)

SI Contents:

[**Figure S1.** – RPLC Gradient Information S2](#_Toc163498888)

[**Figure S2.** – PG Correction Factor S3](#_Toc163498889)

[**Table S1.** – Calibrated ^TW(SLIM)^CCS Values (Beta Prototype) S4](#_Toc163498890)

[**Figure S3.** – DTIM vs. HRIM Heat Maps S10](#_Toc163498891)

[**Figure S4.** – DTIM vs. HRIM Lipid Features S11](#_Toc163498892)

[**Figure S5.** – m/z – CCS Scatter Plots S12](#_Toc163498893)

[**Figure S6.** – HFAP Ion Comparison S14](#_Toc163498894)

[**Figure S7.** – MOBIE Platform Correction Factors S15](#_Toc163498895)

[**Table S2.** – Calibrated Lipid ^TW(SLIM)^CCS Values (MOBIE Platform) S16](#_Toc163498896)

[**Table S3.** – Comparison of Correction Factors S19](#_Toc163498897)

**Figure S1.** RPLC gradient used for SLIM IM-MS data acquisition on the beta prototype and MOBIE platform. RPLC was conducted with a 1290 Infinity II LC system (Agilent). Mobile phases consisted of both 10 mM ammonium formate and 0.1% formic acid in (A) H2O and (B) 60:36:4 IPA:ACN:H2O. RPLC was performed using a C-18 column (HypersilGold 1.9 µm, 2.1 mm x 100 mm, Thermo Fisher) at 40°C with a flow rate of 250 µL/min over a 30-min gradient.

**Figure S2.** CCS calibration bias for the PG subclass calibrated using HFAPs with a 3^rd^ order polynomial as compared to established ^DT^CCS values (0% Bias line). Gray dots show the original bias for calibrated ^TW(SLIM)^CCS values whereas the pink dots show biases for the PG subclass after an applied correction factor of 0.0187. Lipid features selected for calculating the PG correction factor are indicated in Table S1 with an asterisk. Error bars show standard deviation of biases obtained from triplicate inter-day empirical measurements of the arrival times.

**Table S1.** Table of calibrated ^TW(SLIM)^CCS values for all seven total lipid fractions analyzed by the beta prototype SLIM IM-MS. This includes the correction factor for PG shown in Figure S1. Putative IDs are based on accurate mass, retention time (RT), and ^DT^CCS values from the Unified CCS Compendium, and supported by empirical mobility-mass correlations, when available. The bias is the percent difference in ^TW(SLIM)^CCS from ^DT^CCS values. The number of inter-day replicates is represented by “n.”

| **Putative ID** | | | | **Feature Description** | | | | | |
| --- | --- | --- | --- | --- | --- | --- | --- | --- | --- |
| Glycerophosphocholines (PC Egg) | | | | | | | | | |
| Name | Adduct | ^DT^CCS (Å^2^) | Bias (%) | *m/z* (measured) | Peak # | ^TW(SLIM)^CCS (Å^2^) | RSD (%) | RT (min) | n |
| PC 34:03 | [M+H] | 278.2 | 0.27 | 756.5566 | 1 | 278.9 | 0.15 | 18.1 | 4 |
| PC 34:02 | [M+H] | 280.6 | 0.51 | 758.5736 | 1 | 282.0 | 0.23 | 22.8 | 4 |
| PC 34:02 | [M+Na] | 284.2 | -0.50 | 780.5555 | 1 | 282.8 | 0.25 | 15.3 | 3 |
| PC 34:01 | [M+Na] | 286.0 | -0.98 | 782.5732 | 1 | 283.2 | 0.21 | 19.8 | 3 |
| PC 34:01 | [M+Na] | 286.0 | -0.59 | 782.5721 | 2 | 284.3 | 0.11 | 21.5 | 4 |
| PC 36:04 | [M+Na] | 286.2 | -0.39 | 804.5565 | 1 | 285.1 | 0.10 | 14.5 | 3 |
| PC 34:01 | [M+2Na-H] | 285.0 | 1.02 | 804.5550 | 2 | 287.9 | 0.10 | 21.8 | 3 |
| PC 36:03 | [M+Na] | 288.2 | -0.91 | 806.5712 | 1 | 285.6 | 0.29 | 18.4 | 4 |
| PC 34:00 | [M+2Na-H] | 287.2 | 0.14 | 806.5723 | 2 | 287.6 | 0.12 | 18.9 | 3 |
| PC 36:02 | [M+Na] | 289.8 | -0.69 | 808.5867 | 1 | 287.8 | 0.14 | 21.8 | 4 |
| PC 36:02 | [M+Na] | 289.8 | -0.20 | 808.5880 | 2 | 289.2 | 0.11 | 22.8 | 4 |
| PC 36:03 | [M+2Na-H] | 289.6 | 0.33 | 828.5543 | 1 | 290.6 | 0.06 | 18.7 | 3 |
| PC 36:02 | [M+2Na-H] | 291.5 | 0.69 | 830.5737 | 1 | 293.5 | 0.14 | 22.7 | 3 |
| PC 38:03 | [M+Na] | 294.2 | -0.84 | 834.5994 | 1 | 291.7 | 0.14 | 19.4 | 3 |
| PC 38:03 | [M+Na] | 294.2 | -0.43 | 834.6038 | 2 | 292.9 | 0.09 | 22.8 | 4 |
| **Average % bias** | | | -0.17 | **Average % RSD** | | | 0.15 | | |

| **Putative ID** | | | | **Feature Description** | | | | | |
| --- | --- | --- | --- | --- | --- | --- | --- | --- | --- |
| Glycerophosphoethanolamines (PE) | | | | | | | | | |
| Name | Adduct | ^DT^CCS (Å^2^) | Bias (%) | *m/z* (measured) | Peak # | ^TW(SLIM)^CCS (Å^2^) | RSD (%) | RT (min) | n |
| PE 34:02 | [M+H] | 269.5 | -0.13 | 716.5253 | 1 | 269.1 | 0.05 | 12.4 | 3 |
| PE 34:01 | [M+H] | 271.1 | 0.44 | 718.5398 | 1 | 272.3 | 0.06 | 13.3 | 3 |
| PE (O-36:03) | [M+H] | 273.5 | -0.31 | 728.5604 | 1 | 272.6 | 0.08 | 13.1 | 3 |
| PE (O-36:02) | [M+H] | 276.1 | -0.07 | 730.5775 | 1 | 275.9 | 0.06 | 14.2 | 3 |
| PE (O-36:01) | [M+H] | 278.2 | 0.43 | 732.5923 | 1 | 279.4 | 0.11 | 15.2 | 3 |
| PE 34:02 | [M+Na] | 274.1 | 0.00 | 738.5078 | 1 | 274.1 | 0.05 | 12.4 | 3 |
| PE 36:04 | [M+H] | -- | -- | 740.5231 | 1 | 269.1 | 0.10 | 11.5 | 3 |
| PE 34:01 | [M+Na] | 277.1 | -1.53 | 740.5253 | 2 | 272.9 | 0.04 | 12.1 | 3 |
| PE 34:01 | [M+Na] | 277.1 | 0.21 | 740.5221 | 3 | 277.7 | 0.05 | 13.2 | 3 |
| PE 36:03 | [M+H] | 274.4 | -0.83 | 742.5400 | 1 | 272.1 | 0.06 | 12.4 | 3 |
| PE 36:02 | [M+H] | 276.2 | -0.16 | 744.5557 | 1 | 275.7 | 0.06 | 13.4 | 3 |
| PE 36:01 | [M+H] | 278.1 | 0.25 | 746.5713 | 1 | 278.8 | 0.06 | 14.3 | 3 |
| PE 38:06 | [M+H] | -- | -- | 764.5265 | 1 | 275.3 | 0.05 | 11.7 | 3 |
| PE 36:03 | [M+Na] | -- | -- | 764.5242 | 2 | 277.1 | 0.13 | 12.4 | 3 |
| PE 36:02 | [M+Na] | 281.0 | -2.02 | 766.5384 | 1 | 275.3 | 0.08 | 12.0 | 3 |
| PE 38:05 | [M+H] | -- | -- | 766.5412 | 2 | 277.9 | 0.05 | 12.5 | 3 |
| PE 36:02 | [M+Na] | 281.0 | -0.06 | 766.5380 | 3 | 280.7 | 0.08 | 13.4 | 3 |
| PE 38:04 | [M+H] | -- | -- | 768.5558 | 1 | 279.5 | 0.04 | 13.1 | 3 |
| PE 36:01 | [M+Na] | 283.3 | 0.31 | 768.5529 | 2 | 284.0 | 0.08 | 14.3 | 3 |
| PE (O-38:03) | [M+Na] | 284.4 | -0.62 | 778.5768 | 1 | 282.6 | 0.11 | 13.5 | 3 |
| PE (O-38:02) | [M+Na] | -- | -- | 780.5908 | 1 | 284.9 | 0.09 | 14.2 | 3 |
| PE 36:03 | [M+2Na-H] | 280.5 | -1.43 | 786.4997 | 1 | 276.5 | 0.16 | 12.4 | 3 |
| PE 40:09 | [M+H] | -- | -- | 786.5071 | 1 | 282.0 | 0.08 | 11.8 | 3 |
| PE 36:02 | [M+2Na-H] | 282.4 | -0.43 | 788.5213 | 1 | 281.2 | 0.08 | 13.0 | 3 |
| PE 40:08 | [M+H] | -- | -- | 788.5235 | 2 | 284.1 | 0.08 | 12.4 | 3 |
| PE 36:01 | [M+2Na-H] | 284.9 | 0.07 | 790.5383 | 1 | 285.1 | 0.05 | 13.1 | 3 |
| PE 38:03 | [M+Na] | -- | -- | 792.5546 | 1 | 282.0 | 0.04 | 12.8 | 3 |
| PE 38:02 | [M+Na] | -- | -- | 794.5723 | 1 | 284.6 | 0.05 | 13.5 | 3 |
| PE 38:01 | [M+Na] | 287.1 | -0.53 | 796.5852 | 1 | 285.6 | 0.03 | 13.8 | 3 |
| PE 40:07 | [M+Na] | -- | -- | 812.5240 | 1 | 284.0 | 0.12 | 13.1 | 3 |
| PE 40:06 | [M+Na] | -- | -- | 814.5371 | 1 | 288.7 | 0.19 | 12.9 | 3 |
| PE 40:05 | [M+Na] | 290.5 | 0.07 | 816.5500 | 1 | 290.7 | 0.07 | 13.5 | 3 |
| **Average % bias** | | | -0.32 | **Average % RSD** | | | 0.08 | | |

| **Putative ID** | | | | **Feature Description** | | | | | |
| --- | --- | --- | --- | --- | --- | --- | --- | --- | --- |
| Glycerophosphoserines (PS) | | | | | | | | | |
| Name | Adduct | ^DT^CCS (Å^2^) | Bias (%) | *m/z* (measured) | Peak # | ^TW(SLIM)^CCS (Å^2^) | RSD (%) | RT (min) | n |
| PS 36:02 | [M+H] | 283.1 | -0.57 | 788.5498 | 1 | 281.5 | 0.35 | 13.1 | 3 |
| PS 36:01 | [M+H] | 285.6 | -0.09 | 790.5652 | 1 | 285.3 | 0.35 | 14.8 | 3 |
| PS 38:05 | [M+H] | 283.9 | -0.19 | 810.5301 | 1 | 283.4 | 0.46 | 12.8 | 3 |
| PS 38:04 | [M+H] | 286.7 | -0.53 | 812.5486 | 1 | 285.2 | 0.35 | 13.0 | 3 |
| PS 38:04 | [M+H] | 286.7 | 0.08 | 812.5478 | 2 | 286.9 | 0.34 | 14.8 | 3 |
| PS 38:03 | [M+H] | -- | -- | 814.5644 | 1 | 286.4 | 0.35 | 14.4 | 3 |
| PS 38:03 | [M+H] | -- | -- | 814.5634 | 2 | 288.1 | 0.36 | 14.5 | 3 |
| PS 38:02 | [M+H] | 290.0 | -0.82 | 816.5814 | 1 | 287.6 | 0.32 | 15.3 | 3 |
| PS 38:02 | [M+H] | 290.0 | -0.22 | 816.5839 | 2 | 289.4 | 0.34 | 14.5 | 3 |
| PS 38:01 | [M+H] | 291.9 | -0.23 | 818.5971 | 1 | 291.2 | 0.34 | 22.8 | 3 |
| PS 36:01 | [M+2Na-H] | 290.2 | -0.49 | 834.5344 | 1 | 288.8 | 0.31 | 15.0 | 3 |
| PS 40:06 | [M+H] | 290.0 | -0.85 | 836.5498 | 1 | 287.6 | 0.36 | 12.4 | 3 |
| PS 40:05 | [M+H] | 291.9 | -0.60 | 838.5652 | 1 | 290.2 | 0.36 | 13.3 | 3 |
| PS 40:04 | [M+H] | 293.5 | -0.85 | 840.5806 | 1 | 291.0 | 0.35 | 13.9 | 3 |
| PS 40:03 | [M+H] | 294.9 | -0.36 | 842.5969 | 1 | 293.8 | 0.31 | 15.4 | 3 |
| PS 40:02 | [M+H] | 296.3 | -0.74 | 844.6126 | 1 | 294.1 | 0.33 | 19.9 | 3 |
| PS 40:06 | [M+Na] | 294.0 | -0.67 | 858.5312 | 1 | 292.0 | 0.34 | 12.4 | 3 |
| PS 40:05 | [M+Na] | 296.1 | -0.50 | 860.5490 | 1 | 294.6 | 0.35 | 13.4 | 3 |
| PS 40:04 | [M+Na] | 297.6 | -0.83 | 862.5625 | 1 | 295.1 | 0.35 | 14.1 | 3 |
| PS 42:05 | [M+H] | 297.6 | -1.28 | 866.5986 | 1 | 293.8 | 0.38 | 14.7 | 3 |
| PS 42:05 | [M+H] | 297.6 | -0.65 | 866.5954 | 2 | 295.7 | 0.33 | 13.9 | 3 |
| PS 42:04 | [M+H] | 298.7 | -0.84 | 868.6128 | 1 | 296.2 | 0.34 | 22.8 | 3 |
| **Average % bias** | | | -0.56 | **Average % RSD** | | | 0.35 | | |

| **Putative ID** | | | | **Feature Description** | | | | | |
| --- | --- | --- | --- | --- | --- | --- | --- | --- | --- |
| Glucosylceramides (GlcCer) | | | | | | | | | |
| Name | Adduct | ^DT^CCS (Å^2^) | Bias (%) | *m/z* (measured) | Peak # | ^TW(SLIM)^CCS (Å^2^) | RSD (%) | RT (min) | n |
| GlcCer 36:01 | [M+H-H_2_O] | 281.0 | -0.24 | 710.5946 | 1 | 280.3 | 0.25 | 12.8 | 3 |
| GlcCer 36:01 | [M+H-H_2_O] | 281.0 | 0.56 | 710.5929 | 2 | 282.6 | 0.25 | 12.8 | 3 |
| GlcCer 36:02 | [M+H] | 282.5 | -0.57 | 726.5882 | 1 | 280.9 | 0.23 | 12.6 | 3 |
| GlcCer 36:02 | [M+H] | 282.5 | 0.95 | 726.5896 | 2 | 285.2 | 0.28 | 12.6 | 3 |
| GlcCer 36:01 | [M+H] | 283.5 | 0.00 | 728.6097 | 1 | 283.5 | 0.33 | 12.7 | 3 |
| GlcCer 36:00 | [M+H] | 285.7 | -0.03 | 730.6296 | 1 | 285.6 | 0.22 | 13.2 | 3 |
| GlcCer 38:01 | [M+H-H_2_O] | 287.8 | -0.41 | 738.6324 | 1 | 286.6 | 0.24 | 13.7 | 3 |
| GlcCer 38:01 | [M+H-H_2_O] | 287.8 | 0.20 | 738.6310 | 2 | 288.4 | 0.25 | 13.7 | 3 |
| GlcCer 38:02 | [M+H] | 289.0 | -0.59 | 754.6264 | 1 | 287.3 | 0.23 | 13.5 | 3 |
| GlcCer 38:02 | [M+H] | 289.0 | 0.60 | 754.6293 | 2 | 290.7 | 0.26 | 13.5 | 3 |
| GlcCer 38:01 | [M+H] | 289.3 | -0.18 | 756.6385 | 1 | 288.8 | 0.28 | 13.6 | 3 |
| GlcCer 38:00 | [M+H] | 291.3 | -0.18 | 758.6601 | 1 | 290.8 | 0.28 | 14.0 | 3 |
| GlcCer 40:02 | [M+H-H_2_O] | 291.5 | -0.63 | 764.6508 | 1 | 289.7 | 0.22 | 13.7 | 3 |
| GlcCer 40:02 | [M+H-H_2_O] | 291.5 | -0.01 | 764.6476 | 2 | 291.5 | 0.21 | 13.6 | 3 |
| GlcCer 40:01 | [M+H-H_2_O] | 293.9 | -0.49 | 766.6640 | 1 | 292.5 | 0.25 | 14.4 | 3 |
| GlcCer 40:01 | [M+H-H_2_O] | 293.9 | 0.04 | 766.6624 | 2 | 294.0 | 0.28 | 14.5 | 3 |
| GlcCer 38:01 | [M+Na] | 291.7 | -1.11 | 778.6220 | 1 | 288.5 | 0.26 | 13.7 | 3 |
| GlcCer 41:02 | [M+H-H_2_O] | -- | -- | 778.6630 | 1 | 294.6 | 0.22 | 14.0 | 3 |
| GlcCer 38:00 | [M+Na] | 292.4 | -0.62 | 780.6438 | 1 | 290.6 | 0.23 | 13.5 | 3 |
| GlcCer 41:01 | [M+H-H_2_O] | 295.8 | -0.01 | 780.6792 | 1 | 295.8 | 0.24 | 14.8 | 3 |
| GlcCer 41:01 | [M+H-H_2_O] | 295.8 | 0.42 | 780.6799 | 2 | 297.0 | 0.40 | 14.8 | 2 |
| GlcCer 40:02 | [M+H] | 295.1 | -0.62 | 782.6582 | 1 | 293.3 | 0.26 | 14.3 | 3 |
| GlcCer 40:02 | [M+H] | 295.1 | 0.26 | 782.6581 | 2 | 295.9 | 0.25 | 14.3 | 3 |
| GlcCer 40:01 | [M+H] | 295.0 | -0.40 | 784.6758 | 1 | 293.8 | 0.28 | 13.9 | 3 |
| GlcCer 40:00 | [M+H] | 297.4 | -0.36 | 786.6911 | 1 | 296.3 | 0.27 | 14.7 | 3 |
| GlcCer 40:00 | [M+H] | 297.4 | 0.44 | 786.6909 | 2 | 298.7 | 0.31 | 14.7 | 3 |
| GlcCer 42:02 | [M+H-H_2_O] | 297.6 | -0.70 | 792.6798 | 1 | 295.5 | 0.25 | 14.4 | 3 |
| GlcCer 42:02 | [M+H-H_2_O] | 297.6 | -0.13 | 792.6792 | 2 | 297.2 | 0.24 | 14.4 | 3 |
| GlcCer 38:01 OH | [M+Na] | 294.2 | -1.02 | 794.6198 | 1 | 291.2 | 0.26 | 13.5 | 3 |
| GlcCer 42:01 | [M+H-H_2_O] | 299.5 | -0.37 | 794.6954 | 1 | 298.4 | 0.25 | 15.1 | 3 |
| GlcCer 42:01 | [M+H-H_2_O] | 299.5 | 0.09 | 794.6957 | 2 | 299.8 | 0.28 | 15.1 | 3 |
| GlcCer 40:00 OH | [M+H] | 301.4 | -0.75 | 802.6849 | 1 | 299.1 | 0.28 | 14.5 | 3 |
| GlcCer 40:02 | [M+Na] | 293.2 | -0.97 | 804.6381 | 1 | 290.4 | 0.24 | 13.7 | 3 |
| GlcCer 40:01 | [M+Na] | 297.3 | -0.95 | 806.6548 | 1 | 294.5 | 0.27 | 14.4 | 3 |
| GlcCer 40:01 | [M+Na] | 297.3 | -0.39 | 806.6594 | 2 | 296.2 | 0.33 | 13.5 | 3 |
| GlcCer 43:02 | [M+H-H_2_O] | -- | -- | 806.6942 | 1 | 298.6 | 0.22 | 14.7 | 3 |
| GlcCer 43:02 | [M+H-H_2_O] | -- | -- | 806.6955 | 2 | 300.4 | 0.23 | 14.7 | 3 |
| GlcCer 40:00 | [M+Na] | 298.9 | -0.80 | 808.6747 | 1 | 296.5 | 0.27 | 14.2 | 3 |
| GlcCer 40:00 | [M+Na] | 298.9 | 0.00 | 808.6739 | 2 | 298.9 | 0.26 | 14.2 | 3 |
| GlcCer 43:01 | [M+H-H_2_O] | -- | -- | 808.7114 | 1 | 302.1 | 0.17 | 15.4 | 3 |
| GlcCer 42:02 | [M+H] | 300.4 | -0.58 | 810.6820 | 1 | 298.7 | 0.16 | 14.3 | 2 |
| GlcCer 42:02 | [M+H] | 300.4 | -0.40 | 810.6909 | 2 | 299.2 | 0.26 | 15.0 | 3 |
| GlcCer 42:02 | [M+H] | 300.4 | 0.33 | 810.6900 | 3 | 301.4 | 0.26 | 15.0 | 3 |
| GlcCer 42:00 | [M+H] | 303.5 | -0.51 | 814.7215 | 1 | 301.9 | 0.28 | 15.3 | 3 |
| GlcCer 41:02 | [M+Na] | 297.3 | -1.19 | 818.6575 | 1 | 293.8 | 0.30 | 14.1 | 3 |
| GlcCer 41:01 | [M+Na] | 298.6 | -0.29 | 820.6707 | 1 | 297.7 | 0.28 | 14.8 | 3 |
| GlcCer 44:02 | [M+H-H_2_O] | 302.7 | -0.36 | 820.7105 | 1 | 301.6 | 0.26 | 15.1 | 3 |
| GlcCer 44:02 | [M+H-H_2_O] | 302.7 | 0.19 | 820.7118 | 2 | 303.3 | 0.25 | 15.1 | 3 |
| GlcCer 42:04 OH | [M+H] | 300.2 | -1.05 | 822.6510 | 1 | 297.1 | 0.27 | 14.3 | 3 |
| GlcCer 43:03 | [M+H] | -- | -- | 822.6897 | 1 | 299.5 | 0.26 | 14.6 | 3 |
| GlcCer 43:02 OH | [M+H-H_2_O] | -- | -- | 822.6901 | 2 | 301.8 | 0.25 | 14.6 | 3 |
| GlcCer 44:01 | [M+H-H_2_O] | -- | -- | 822.7263 | 1 | 305.2 | 0.23 | 15.7 | 3 |
| GlcCer 42:03 OH | [M+H] | 301.6 | -1.02 | 824.6731 | 1 | 298.5 | 0.23 | 14.5 | 3 |
| GlcCer 43:02 | [M+H] | 303.2 | -0.36 | 824.7059 | 1 | 302.1 | 0.26 | 15.3 | 3 |
| GlcCer 43:02 | [M+H] | 303.2 | 0.32 | 824.7070 | 2 | 304.2 | 0.25 | 15.3 | 3 |
| GlcCer 42:01 OH | [M+H] | 305.9 | -1.00 | 828.7004 | 1 | 302.9 | 0.24 | 14.5 | 3 |
| GlcCer 42:00 OH | [M+H] | 307.3 | -0.83 | 830.7162 | 1 | 304.8 | 0.26 | 15.2 | 3 |
| GlcCer 42:02 | [M+Na] | 300.7 | -1.20 | 832.6720 | 1 | 297.1 | 0.28 | 14.4 | 3 |
| GlcCer 42:01 | [M+Na] | 302.9 | -1.47 | 834.6881 | 1 | 298.5 | 0.25 | 14.6 | 3 |
| GlcCer 42:01 | [M+Na] | 302.9 | -0.71 | 834.6888 | 2 | 300.8 | 0.26 | 15.1 | 3 |
| GlcCer 42:00 | [M+Na] | 304.3 | -0.59 | 836.7055 | 1 | 302.5 | 0.26 | 14.9 | 3 |
| GlcCer 42:00 | [M+Na] | 304.3 | 0.12 | 836.7059 | 2 | 304.7 | 0.26 | 14.9 | 3 |
| GlcCer 42:00 | [M+Na] | 304.3 | 0.77 | 836.7067 | 3 | 306.7 | 0.23 | 14.9 | 3 |
| GlcCer 44:02 | [M+H] | 306.3 | -0.41 | 838.7204 | 1 | 305.1 | 0.25 | 15.6 | 3 |
| GlcCer 44:02 | [M+H] | 306.3 | 0.30 | 838.7213 | 2 | 307.2 | 0.20 | 15.6 | 3 |
| GlcCer 44:01 | [M+H] | 307.6 | -0.68 | 840.7385 | 1 | 305.5 | 0.27 | 15.3 | 3 |
| GlcCer 47:03 | [M+H-2H_2_O] | 308.9 | -0.45 | 842.7286 | 1 | 307.5 | 0.26 | 14.5 | 3 |
| GlcCer 43:00 OH | [M+H] | -- | -- | 844.7331 | 1 | 307.5 | 0.25 | 15.5 | 3 |
| GlcCer 42:03 OH | [M+Na] | -- | -- | 846.6504 | 1 | 296.4 | 0.27 | 13.5 | 3 |
| GlcCer 43:02 | [M+Na] | 303.6 | -1.03 | 846.6902 | 1 | 300.5 | 0.24 | 14.8 | 3 |
| GlcCer 42:02 OH | [M+Na] | 303.1 | -1.28 | 848.6671 | 1 | 299.2 | 0.28 | 14.2 | 3 |
| GlcCer 42:01 OH | [M+Na] | 305.4 | -0.76 | 850.6822 | 1 | 303.1 | 0.26 | 14.9 | 3 |
| GlcCer 42:00 OH | [M+Na] | 306.7 | -0.72 | 852.6981 | 1 | 304.5 | 0.26 | 15.2 | 3 |
| GlcCer 44:02 OH | [M+H] | 310.2 | -1.11 | 854.7153 | 1 | 306.8 | 0.21 | 14.8 | 3 |
| GlcCer 44:01 OH | [M+H] | 311.8 | -1.16 | 856.7337 | 1 | 308.2 | 0.02 | 15.1 | 3 |
| GlcCer 44:01 OH | [M+H] | 311.8 | -1.14 | 856.7319 | 2 | 308.3 | 0.36 | 15.8 | 3 |
| GlcCer 47:04 | [M+H-H_2_O] | -- | -- | 858.7246 | 1 | 309.3 | 0.24 | 14.2 | 3 |
| GlcCer 44:00 OH | [M+H] | 313.1 | -0.89 | 858.7499 | 1 | 310.3 | 0.27 | 15.8 | 3 |
| GlcCer 44:02 | [M+Na] | 307.5 | -1.22 | 860.7051 | 1 | 303.8 | 0.24 | 15.1 | 3 |
| GlcCer 47:03 | [M+H-H_2_O] | -- | -- | 860.7348 | 1 | 310.5 | 0.20 | 14.6 | 3 |
| GlcCer 43:02 OH | [M+Na] | 307.3 | -1.52 | 862.6834 | 1 | 302.7 | 0.26 | 14.6 | 3 |
| GlcCer 44:01 | [M+Na] | 309.1 | -1.30 | 862.7153 | 1 | 305.1 | 0.37 | 15.2 | 3 |
| GlcCer 43:01 OH | [M+Na] | 307.8 | -0.57 | 864.7027 | 1 | 306.0 | 0.29 | 15.3 | 3 |
| GlcCer 46:03 | [M+H] | -- | -- | 864.7367 | 1 | 308.4 | 0.23 | 15.5 | 3 |
| GlcCer 47:00 | [M+Na-2H_2_O] | 314.8 | -0.80 | 870.7605 | 1 | 312.3 | 0.26 | 14.6 | 3 |
| GlcCer 47:00 | [M+Na-2H_2_O] | 314.8 | -0.43 | 870.7589 | 2 | 313.5 | 0.28 | 15.1 | 3 |
| GlcCer 45:00 OH | [M+H] | 316.3 | -1.42 | 872.7603 | 1 | 311.9 | 0.06 | 14.6 | 2 |
| GlcCer 46:05 OH | [M+H] | 309.9 | -1.31 | 876.6985 | 1 | 305.9 | 0.26 | 14.9 | 3 |
| GlcCer 46:04 OH | [M+H] | 310.7 | -0.50 | 878.7136 | 1 | 309.2 | 0.25 | 15.6 | 3 |
| GlcCer 50:04 | [M+H-2H_2_O] | 315.5 | -0.77 | 882.7616 | 1 | 313.1 | 0.05 | 14.7 | 2 |
| GlcCer 46:02 | [M+H-H_2_O] | 315.5 | -1.01 | 884.7373 | 1 | 312.3 | 0.27 | 14.3 | 3 |
| GlcCer 46:01 | [M+H-H_2_O] | 317.5 | -0.75 | 886.7552 | 1 | 315.1 | 0.26 | 15.0 | 3 |
| GlcCer 49:01 | [M+Na-2H_2_O] | 318.8 | -0.74 | 896.7752 | 1 | 316.5 | 0.26 | 15.1 | 3 |
| GlcCer 49:00 | [M+Na-2H_2_O] | 320.0 | -1.19 | 898.7962 | 1 | 316.2 | 0.23 | 16.6 | 3 |
| **Average % bias** | | | -0.51 | **Average % RSD** | | | 0.25 | | |

| **Putative ID** | | | | **Feature Description** | | | | | |
| --- | --- | --- | --- | --- | --- | --- | --- | --- | --- |
| Sphingomyelins (SM) | | | | | | | | | |
| Name | Adduct | ^DT^CCS (Å^2^) | Bias (%) | *m/z* (measured) | Peak # | ^TW(SLIM)^CCS (Å^2^) | RSD (%) | RT (min) | n |
| SM 34:01 | [M+H] | 281.2 | -0.42 | 703.5804 | 1 | 280.0 | 0.28 | 18.5 | 3 |
| SM 36:02 | [M+H] | 285.3 | -1.03 | 729.5954 | 1 | 282.4 | 0.35 | 19.6 | 2 |
| SM 36:02 | [M+H] | 285.3 | 0.07 | 729.5923 | 2 | 285.5 | 0.37 | 19.4 | 2 |
| SM 36:01 | [M+H] | 288.4 | -0.89 | 731.6065 | 1 | 285.9 | 0.44 | 22.9 | 3 |
| SM 38:01 | [M+H] | 293.4 | -0.66 | 759.6437 | 1 | 291.5 | 0.30 | 10.8 | 3 |
| SM 39:01 | [M+H] | 297.0 | -0.93 | 773.6580 | 1 | 294.3 | 0.28 | 10.4 | 3 |
| SM 40:02 | [M+H] | 296.8 | -0.88 | 785.6589 | 1 | 294.2 | 0.27 | 12.0 | 3 |
| SM 40:01 | [M+H] | 299.1 | -0.69 | 787.6732 | 1 | 297.0 | 0.29 | 12.2 | 3 |
| SM 40:00 | [M+H] | 300.8 | -0.45 | 789.6895 | 1 | 299.4 | 0.28 | 14.9 | 3 |
| SM 39:01 | [M+Na] | 295.7 | -0.64 | 795.6431 | 1 | 293.8 | 0.04 | 11.2 | 2 |
| SM 41:02 | [M+H] | 300.1 | -0.93 | 799.6745 | 1 | 297.3 | 0.26 | 10.4 | 3 |
| SM 41:01 | [M+H] | 302.3 | -0.85 | 801.6890 | 1 | 299.7 | 0.29 | 16.1 | 3 |
| SM 41:00 | [M+H] | 303.5 | -0.53 | 803.7047 | 1 | 301.9 | 0.29 | 21.5 | 3 |
| SM 42:04 | [M+H] | 299.0 | -0.60 | 809.6564 | 1 | 297.2 | 0.32 | 12.9 | 3 |
| SM 42:03 | [M+H] | 300.8 | -0.85 | 811.6725 | 1 | 298.2 | 0.23 | 15.1 | 3 |
| SM 42:02 | [M+H] | 302.2 | -0.81 | 813.6903 | 1 | 299.7 | 0.30 | 13.4 | 3 |
| SM 42:01 | [M+H] | 304.4 | -0.63 | 815.7049 | 1 | 302.5 | 0.31 | 22.7 | 3 |
| SM 42:00 | [M+H] | 306.5 | -0.54 | 817.7108 | 1 | 304.9 | 0.39 | 20.6 | 2 |
| SM 41:01 | [M+Na] | 300.5 | -0.22 | 823.6716 | 1 | 299.8 | 0.30 | 17.6 | 3 |
| SM 43:03 | [M+H] | -- | -- | 825.6831 | 1 | 300.9 | 0.30 | 13.2 | 3 |
| SM 43:02 | [M+H] | 305.7 | -1.05 | 827.7056 | 1 | 302.5 | 0.27 | 16.0 | 3 |
| SM 43:01 | [M+H] | 308.1 | -0.98 | 829.7178 | 1 | 305.1 | 0.32 | 17.2 | 3 |
| SM 42:01 | [M+Na] | 303.8 | -0.46 | 837.6894 | 1 | 302.4 | 0.36 | 17.9 | 3 |
| SM 44:02 | [M+H] | 308.7 | -1.06 | 841.7234 | 1 | 305.4 | 0.28 | 19.4 | 3 |
| **Average % bias** | | | -0.70 | **Average % RSD** | | | 0.30 | | |

| **Putative ID** | | | | **Feature Description** | | | | | |
| --- | --- | --- | --- | --- | --- | --- | --- | --- | --- |
| Glycerophosphocholines (PC Soy) | | | | | | | | | |
| Name | Adduct | ^DT^CCS (Å^2^) | Bias (%) | *m/z* (measured) | Peak # | ^TW(SLIM)^CCS (Å^2^) | RSD (%) | RT (min) | n |
| PC 32:01 | [M+H] | 277.6 | -5.33 | 732.5513 | 1 | 263.2 | 0.11 | 16.4 | 3 |
| PC 34:03 | [M+H] | 278.2 | -0.45 | 756.5569 | 1 | 276.9 | 0.14 | 17.0 | 3 |
| PC 34:02 | [M+H] | 280.6 | -0.22 | 758.5715 | 1 | 280.0 | 0.17 | 22.7 | 2 |
| PC 34:03 | [M+Na] | 283.1 | -3.07 | 778.5392 | 1 | 274.5 | 0.10 | 12.0 | 3 |
| PC 34:03 | [M+Na] | 283.1 | -1.31 | 778.5395 | 2 | 279.4 | 0.11 | 17.1 | 3 |
| PC 34:02 | [M+Na] | 284.2 | -2.41 | 780.5546 | 1 | 277.4 | 0.12 | 13.6 | 3 |
| PC 34:02 | [M+Na] | 284.2 | -0.73 | 780.5535 | 2 | 282.1 | 0.16 | 22.9 | 2 |
| PC 34:01 | [M+Na] | 286.0 | -2.01 | 782.5707 | 1 | 280.3 | 0.13 | 16.2 | 3 |
| PC 34:00 | [M+Na] | -- | -- | 784.5870 | 1 | 283.5 | 0.17 | 22.8 | 2 |
| PC 34:02 | [M+2Na-H] | -- | -- | 802.5377 | 1 | 279.8 | 0.13 | 13.4 | 3 |
| PC 34:01 | [M+2Na-H] | 285.0 | -0.84 | 804.5539 | 1 | 282.6 | 0.13 | 16.1 | 3 |
| PC 34:00 | [M+2Na-H] | 287.2 | -0.46 | 806.5694 | 1 | 285.9 | 0.17 | 22.8 | 2 |
| **Average % bias** | | | -1.68 | **Average % RSD** | | | 0.14 | | |

| **Putative ID** | | | | **Feature Description** | | | | | |
| --- | --- | --- | --- | --- | --- | --- | --- | --- | --- |
| Glycerophosphoglycerols (PG) | | | | | | | | | |
| Name | Adduct | ^DT^CCS (Å^2^) | Bias (%) | *m/z* (measured) | Peak # | ^TW(SLIM)^CCS (Å^2^) | RSD (%) | RT (min) | n |
| *PG 34:02 | [M+Na] | 277.4 | 0.28 | 769.5028 | 1 | 278.2 | 0.36 | 11.0 | 3 |
| *PG 34:01 | [M+Na] | 279.3 | 0.72 | 771.5186 | 1 | 281.3 | 0.33 | 11.8 | 3 |
| *PG 34:02 | [M+2Na-H] | 279.2 | -0.31 | 791.4859 | 1 | 278.3 | 0.35 | 11.0 | 3 |
| PG O-40:06 | [M+H-H_2_O] | -- | -- | 791.5576 | 1 | 289.8 | 0.34 | 11.0 | 3 |
| PG 34:01 | [M+2Na-H] | 280.5 | -0.05 | 793.5003 | 1 | 280.4 | 0.33 | 11.9 | 3 |
| PG 34:01 | [M+2Na-H] | 280.5 | 0.57 | 793.5027 | 2 | 282.1 | 0.37 | 10.8 | 3 |
| PG O-40:05 | [M+H-H_2_O] | -- | -- | 793.5756 | 1 | 293.8 | 0.35 | 11.9 | 3 |
| *PG 36:04 | [M+2Na-H] | 283.6 | -0.42 | 815.4841 | 1 | 282.4 | 0.33 | 10.8 | 3 |
| PG O-42:08 | [M+H-H_2_O] | -- | -- | 815.5639 | 1 | 292.5 | 0.34 | 10.8 | 3 |
| PG 36:03 | [M+2Na-H] | 285.0 | -0.54 | 817.4937 | 1 | 283.5 | 0.73 | 11.4 | 2 |
| PG 36:03 | [M+2Na-H] | 285.0 | 0.02 | 817.5018 | 2 | 285.0 | 0.34 | 10.5 | 3 |
| PG O-42:07 | [M+H-H_2_O] | -- | -- | 817.5710 | 1 | 292.8 | 0.34 | 11.2 | 3 |
| PG 36:02 | [M+2Na-H] | 286.6 | -0.42 | 819.5149 | 1 | 285.4 | 0.34 | 12.1 | 3 |
| PG 36:02 | [M+2Na-H] | 286.6 | 0.42 | 819.5186 | 2 | 287.8 | 0.38 | 11.2 | 3 |
| PG O-42:06 | [M+H-H_2_O] | -- | -- | 819.5892 | 1 | 296.0 | 0.36 | 12.1 | 3 |
| PG 36:01 | [M+2Na-H] | 288.2 | -0.24 | 821.5328 | 1 | 287.5 | 0.33 | 12.9 | 3 |
| PG 36:01 | [M+2Na-H] | 288.2 | 0.20 | 821.5344 | 2 | 288.8 | 0.32 | 11.7 | 3 |
| PG O-42:05 | [M+H-H_2_O] | -- | -- | 821.6045 | 1 | 299.7 | 0.34 | 12.8 | 3 |
| *PG 38:05 | [M+2Na-H] | 289.1 | -0.15 | 841.4995 | 1 | 288.7 | 0.48 | 11.2 | 2 |
| PG O-44:09 | [M+H-H_2_O] | -- | -- | 841.5738 | 1 | 297.3 | 0.39 | 11.2 | 3 |
| *PG 38:04 | [M+2Na-H] | 290.2 | -0.23 | 843.5167 | 1 | 289.5 | 0.50 | 11.7 | 2 |
| PG O-44:08 | [M+H-H_2_O] | -- | -- | 843.5924 | 1 | 298.5 | 0.34 | 11.7 | 3 |
| **Average % bias** | | | -0.01 | **Average % RSD** | | | 0.38 | | |

**Figure S3.** (Left) DTIM-MS heat map of PE 34:01 [M+Na]^+^ at *m/z* 740.52. Only one IM feature is shown at this specific mass-to-charge. (Right) SLIM IM-MS heat map of the same lipid feature, but now showing three resulting IM features at the same mass-to-charge.

**Figure S4.** Comparison of the total number of lipid features observed with both DTIM and HRIM (beta prototype). For each lipid subclass fraction, the HRIM total lipid features is higher than the DTIM total lipid features since the HRIM platform achieves higher resolving powers without a loss in sensitivity, allowing for the potential resolving of a single DTIM feature into multiple lipid features.

**Figure S5.** Mobility-mass correlation plots for each total lipid fraction. Chain length and unsaturation trendlines (n ≥ 3 data points) are indicated at the left and right columns, respectively.

**Figure S6.** Agilent HFAP ion arrival times comparison of the SLIM IM beta prototype and MOBIE platform operated with the same SLIM IM parameters (WH = 40 V_pp_, WV = 180 m/s).

**Figure S7.** CCS bias corresponding to using only the 3^rd^ order polynomial calibration (gray) vs. using the calibration with updated linear correction factors (color) for the MOBIE platform data from four selected lipid subclasses (PC, PE, GlcCer, and PG) are shown. Lipid features chosen within each subclass exhibited single peaks in both IM and HRIM and spanned the subclass *m/z* range. Lipid features selected for calculating the updated subclass correction factor are indicated in Table S2 with an asterisk. Error bars are present for each data point, but they are often within the size of the marker.

**Table S2.** Table of calibrated ^TW(SLIM)^CCS values for five lipid extracts analyzed by the MOBIE platform. These incorporate the correction factors shown in Figure S7. Retention times omitted (refer to Table S1).

| **Putative ID** | | | | **Feature Description** | | | | |
| --- | --- | --- | --- | --- | --- | --- | --- | --- |
| Glycerophosphocholines (PC Egg) | | | | | | | | |
| Name | Adduct | ^DT^CCS (Å^2^) | Bias (%) | *m/z* (measured) | Peak # | ^TW(SLIM)^CCS (Å^2^) | RSD (%) | n |
| *PC 34:03 | [M+H] | 278.2 | 1.27 | 756.5566 | 1 | 281.8 | 0.08 | 2 |
| *PC 34:02 | [M+Na] | 284.2 | 0.21 | 780.5555 | 1 | 284.8 | 0.10 | 2 |
| PC 34:01 | [M+Na] | 286.0 | -0.44 | 782.5721 | 1 | 284.7 | 0.00 | 2 |
| PC 34:01 | [M+Na] | 286.0 | 0.43 | 782.5732 | 2 | 287.2 | 0.00 | 2 |
| *PC 36:04 | [M+Na] | 286.2 | 1.29 | 804.5565 | 1 | 289.9 | 0.04 | 2 |
| *PC 36:03 | [M+Na] | 288.2 | 0.50 | 806.5712 | 1 | 289.7 | 0.00 | 2 |
| *PC 36:02 | [M+Na] | 289.8 | 0.29 | 808.5867 | 1 | 290.6 | 0.21 | 2 |
| *PC 36:03 | [M+2Na-H] | 289.6 | 1.10 | 828.5543 | 1 | 292.8 | 0.00 | 2 |
| **Average % bias** | | | 0.58 | **Average % RSD** | | | 0.05 | |

| **Putative ID** | | | | **Feature Description** | | | | |
| --- | --- | --- | --- | --- | --- | --- | --- | --- |
| Glycerophosphocholines (PC Soy) | | | | | | | | |
| Name | Adduct | ^DT^CCS (Å^2^) | Bias (%) | *m/z* (measured) | Peak # | ^TW(SLIM)^CCS (Å^2^) | RSD (%) | n |
| PC 32:01 | [M+H] | 277.6 | -4.76 | 732.5513 | 1 | 264.7 | 0.06 | 3 |
| PC 34:03 | [M+H] | 278.2 | 0.70 | 756.5569 | 1 | 280.2 | 0.05 | 3 |
| PC 34:03 | [M+Na] | 283.1 | -2.13 | 778.5392 | 1 | 277.1 | 0.06 | 3 |
| PC 34:03 | [M+Na] | 283.1 | -0.25 | 778.5395 | 2 | 282.4 | 0.11 | 3 |
| PC 34:02 | [M+Na] | 284.2 | -1.38 | 780.5546 | 1 | 280.3 | 0.06 | 3 |
| PC 34:01 | [M+Na] | 286.0 | -0.92 | 782.5707 | 1 | 283.4 | 0.06 | 3 |
| PC 34:02 | [M+2Na-H] | -- | -- | 802.5377 | 1 | 282.3 | 0.08 | 3 |
| PC 34:01 | [M+2Na-H] | 285.0 | 0.12 | 804.5539 | 1 | 285.4 | 0.06 | 3 |
| **Average % bias** | | | -1.23 | **Average % RSD** | | | 0.07 | |

| **Putative ID** | | | | **Feature Description** | | | | |
| --- | --- | --- | --- | --- | --- | --- | --- | --- |
| Glycerophosphoethanolamines (PE) | | | | | | | | |
| Name | Adduct | ^DT^CCS (Å^2^) | Bias (%) | *m/z* (measured) | Peak # | ^TW(SLIM)^CCS (Å^2^) | RSD (%) | n |
| *PE 34:02 | [M+H] | 269.5 | -0.15 | 716.5253 | 1 | 269.1 | 0.07 | 3 |
| *PE 34:01 | [M+H] | 271.1 | 0.60 | 718.5398 | 1 | 272.7 | 0.09 | 3 |
| *PE 34:02 | [M+Na] | 274.1 | 0.01 | 738.5078 | 1 | 274.1 | 0.04 | 3 |
| PE 34:01 | [M+Na] | 277.1 | 0.37 | 740.5221 | 1 | 278.1 | 0.02 | 3 |
| PE 34:01 | [M+Na] | 277.1 | -1.52 | 740.5253 | 2 | 272.9 | 0.02 | 3 |
| *PE 36:03 | [M+H] | 274.4 | -0.83 | 742.5400 | 1 | 272.1 | 0.09 | 3 |
| *PE 36:02 | [M+H] | 276.2 | -0.09 | 744.5557 | 1 | 276.0 | 0.07 | 3 |
| *PE 36:01 | [M+H] | 278.1 | 0.46 | 746.5713 | 1 | 279.4 | 0.06 | 3 |
| PE 36:03 | [M+Na] | -- | -- | 764.5242 | 1 | 275.2 | 0.07 | 3 |
| PE 36:02 | [M+Na] | 281.0 | 0.06 | 766.5380 | 1 | 281.2 | 0.07 | 3 |
| PE 36:02 | [M+Na] | 281.0 | -1.65 | 766.5384 | 2 | 276.4 | 0.04 | 2 |
| PE 38:05 | [M+H] | -- | -- | 766.5412 | 3 | 278.0 | 0.01 | 3 |
| PE 36:01 | [M+Na] | 283.3 | 0.53 | 768.5529 | 1 | 284.8 | 0.06 | 3 |
| PE 38:04 | [M+H] | -- | -- | 768.5558 | 2 | 279.7 | 0.02 | 3 |
| *PE (O-38:03) | [M+Na] | 284.4 | -0.49 | 778.5768 | 1 | 283.0 | 0.06 | 3 |
| PE (O-38:02) | [M+Na] | -- | -- | 780.5908 | 1 | 285.0 | 0.02 | 3 |
| PE 36:02 | [M+2Na-H] | 282.4 | 0.94 | 788.5213 | 1 | 285.1 | 0.01 | 2 |
| *PE 36:01 | [M+2Na-H] | 284.9 | 0.16 | 790.5383 | 1 | 285.4 | 0.04 | 3 |
| PE 38:03 | [M+Na] | -- | -- | 792.5546 | 1 | 282.2 | 0.10 | 3 |
| PE 38:02 | [M+Na] | -- | -- | 794.5723 | 1 | 284.7 | 0.08 | 3 |
| PE 40:07 | [M+Na] | -- | -- | 812.5240 | 1 | 284.0 | 0.24 | 3 |
| *PE 40:05 | [M+Na] | 290.5 | 0.25 | 816.5500 | 1 | 291.2 | 0.01 | 3 |
| **Average % bias** | | | -0.09 | **Average % RSD** | | | 0.06 | |

| **Putative ID** | | | | **Feature Description** | | | | |
| --- | --- | --- | --- | --- | --- | --- | --- | --- |
| Glucosylceramides (GlcCer) | | | | | | | | |
| Name | Adduct | ^DT^CCS (Å^2^) | Bias (%) | *m/z* (measured) | Peak # | ^TW(SLIM)^CCS (Å^2^) | RSD (%) | n |
| GlcCer 36:01 | [M+H-H_2_O] | 281.0 | 0.26 | 710.5946 | 1 | 281.7 | 0.08 | 2 |
| GlcCer 36:01 | [M+H-H_2_O] | 281.0 | 1.02 | 710.5929 | 2 | 283.9 | 0.03 | 2 |
| GlcCer 36:02 | [M+H] | 282.5 | -0.02 | 726.5882 | 1 | 282.4 | 0.11 | 2 |
| GlcCer 36:02 | [M+H] | 282.5 | 1.27 | 726.5896 | 2 | 286.1 | 0.10 | 2 |
| *GlcCer 38:01 | [M+H-H_2_O] | 287.8 | 0.72 | 738.6324 | 1 | 289.9 | 0.15 | 2 |
| GlcCer 38:02 | [M+H] | 289.0 | -0.01 | 754.6264 | 1 | 289.0 | 0.05 | 2 |
| GlcCer 38:02 | [M+H] | 289.0 | 1.07 | 754.6293 | 2 | 292.1 | 0.12 | 2 |
| GlcCer 40:01 | [M+H-H_2_O] | 293.9 | 0.29 | 766.6640 | 1 | 294.7 | 0.00 | 2 |
| GlcCer 38:01 | [M+Na] | 291.7 | -0.45 | 778.6220 | 1 | 290.4 | 0.05 | 2 |
| GlcCer 41:02 | [M+H-H_2_O] | -- | -- | 778.6630 | 1 | 294.7 | 0.04 | 2 |
| GlcCer 38:00 | [M+Na] | 292.4 | -0.03 | 780.6438 | 1 | 292.3 | 0.02 | 2 |
| GlcCer 41:01 | [M+H-H_2_O] | 295.8 | 0.65 | 780.6792 | 1 | 297.7 | 0.18 | 2 |
| GlcCer 40:02 | [M+H] | 295.1 | 0.11 | 782.6582 | 1 | 295.4 | 0.04 | 2 |
| GlcCer 40:02 | [M+H] | 295.1 | 0.98 | 782.6581 | 2 | 298.0 | 0.02 | 2 |
| *GlcCer 40:01 | [M+H] | 295.0 | 0.87 | 784.6758 | 1 | 297.6 | 0.06 | 2 |
| GlcCer 42:02 | [M+H-H_2_O] | 297.6 | 0.13 | 792.6798 | 1 | 298.0 | 0.01 | 2 |
| GlcCer 42:01 | [M+H-H_2_O] | 299.5 | 0.52 | 794.6954 | 1 | 301.1 | 0.11 | 2 |
| GlcCer 40:01 | [M+Na] | 297.3 | -0.11 | 806.6594 | 1 | 297.0 | 0.08 | 2 |
| GlcCer 43:02 | [M+H-H_2_O] | -- | -- | 806.6942 | 1 | 301.0 | 0.00 | 2 |
| GlcCer 40:00 | [M+Na] | 298.9 | -0.11 | 808.6747 | 1 | 298.6 | 0.06 | 2 |
| GlcCer 40:00 | [M+Na] | 298.9 | 0.66 | 808.6739 | 2 | 300.9 | 0.01 | 2 |
| GlcCer 42:02 | [M+H] | 300.4 | 0.36 | 810.6820 | 1 | 301.5 | 0.06 | 2 |
| GlcCer 42:02 | [M+H] | 300.4 | 1.05 | 810.6909 | 2 | 303.6 | 0.04 | 2 |
| *GlcCer 41:02 | [M+Na] | 297.3 | -0.61 | 818.6575 | 1 | 295.5 | 0.04 | 2 |
| GlcCer 41:01 | [M+Na] | 298.6 | 0.38 | 820.6707 | 1 | 299.7 | 0.04 | 2 |
| GlcCer 44:02 | [M+H-H_2_O] | 302.7 | 0.44 | 820.7105 | 1 | 304.0 | 0.00 | 2 |
| GlcCer 42:04 OH | [M+H] | 300.2 | -0.30 | 822.6510 | 1 | 299.3 | 0.04 | 2 |
| GlcCer 43:03 | [M+H] | -- | -- | 822.6897 | 1 | 301.2 | 0.08 | 2 |
| GlcCer 43:02 | [M+H] | 303.2 | 0.37 | 824.7059 | 1 | 304.3 | 0.04 | 2 |
| GlcCer 43:02 | [M+H] | 303.2 | 1.05 | 824.7070 | 2 | 306.4 | 0.08 | 2 |
| *GlcCer 42:02 | [M+Na] | 300.7 | -0.56 | 832.6720 | 1 | 299.0 | 0.05 | 2 |
| GlcCer 42:01 | [M+Na] | 302.9 | -0.84 | 834.6881 | 1 | 300.4 | 0.03 | 2 |
| GlcCer 42:01 | [M+Na] | 302.9 | 0.06 | 834.6888 | 2 | 303.1 | 0.07 | 2 |
| GlcCer 42:00 | [M+Na] | 304.3 | 0.15 | 836.7055 | 1 | 304.8 | 0.01 | 2 |
| GlcCer 42:00 | [M+Na] | 304.3 | 0.81 | 836.7059 | 2 | 306.8 | 0.09 | 2 |
| GlcCer 44:02 | [M+H] | 306.3 | 0.91 | 838.7204 | 1 | 309.1 | 0.01 | 2 |
| GlcCer 43:02 | [M+Na] | 303.6 | -0.40 | 846.6902 | 1 | 302.4 | 0.05 | 2 |
| *GlcCer 42:02 OH | [M+Na] | 303.1 | -0.68 | 848.6671 | 1 | 301.1 | 0.02 | 2 |
| *GlcCer 42:01 OH | [M+Na] | 305.4 | -0.03 | 850.6822 | 1 | 305.3 | 0.04 | 2 |
| *GlcCer 42:00 OH | [M+Na] | 306.7 | 0.01 | 852.6981 | 1 | 306.7 | 0.05 | 2 |
| GlcCer 44:02 | [M+Na] | 307.5 | -0.53 | 860.7051 | 1 | 305.9 | 0.10 | 2 |
| GlcCer 43:02 OH | [M+Na] | 307.3 | -0.85 | 862.6834 | 1 | 304.7 | 0.02 | 2 |
| GlcCer 44:01 | [M+Na] | 309.1 | -0.56 | 862.7153 | 1 | 307.4 | 0.18 | 2 |
| GlcCer 43:01 OH | [M+Na] | 307.8 | 0.23 | 864.7027 | 1 | 308.5 | 0.08 | 2 |
| *GlcCer 46:05 OH | [M+H] | 309.9 | -0.69 | 876.6985 | 1 | 307.8 | 0.01 | 2 |
| *GlcCer 46:04 OH | [M+H] | 310.7 | 0.21 | 878.7136 | 1 | 311.3 | 0.08 | 2 |
| **Average % bias** | | | 0.18 | **Average % RSD** | | | 0.06 | |

| **Putative ID** | | | | **Feature Description** | | | | |
| --- | --- | --- | --- | --- | --- | --- | --- | --- |
| Glycerophosphoglycerols (PG) | | | | | | | | |
| Name | Adduct | ^DT^CCS (Å^2^) | Bias (%) | *m/z* (measured) | Peak # | ^TW(SLIM)^CCS (Å^2^) | RSD (%) | n |
| *PG 34:02 | [M+Na] | 277.4 | 0.11 | 769.5028 | 1 | 277.7 | 0.07 | 3 |
| *PG 34:01 | [M+Na] | 279.3 | 0.71 | 771.5186 | 1 | 281.3 | 0.10 | 3 |
| *PG 34:02 | [M+2Na-H] | 279.2 | -0.55 | 791.4859 | 1 | 277.7 | 0.09 | 3 |
| PG O-40:06 | [M+H-H_2_O] | -- | -- | 791.5576 | 1 | 289.7 | 0.11 | 3 |
| *PG 34:01 | [M+2Na-H] | 280.5 | 0.24 | 793.5003 | 1 | 281.2 | 0.17 | 3 |
| PG O-40:05 | [M+H-H_2_O] | -- | -- | 793.5756 | 1 | 294.8 | 0.07 | 2 |
| PG O-42:08 | [M+H-H_2_O] | -- | -- | 815.5639 | 1 | 293.1 | 0.03 | 2 |
| PG 40:09 | [M+H] | -- | -- | 817.5018 | 1 | 284.4 | 0.03 | 3 |
| PG O-42:07 | [M+H-H_2_O] | -- | -- | 817.5710 | 1 | 293.6 | 0.13 | 2 |
| *PG 36:02 | [M+2Na-H] | 286.6 | -0.66 | 819.5149 | 1 | 284.7 | 0.09 | 3 |
| PG 36:02 | [M+2Na-H] | 286.6 | 0.26 | 819.5186 | 2 | 287.3 | 0.10 | 3 |
| PG O-42:06 | [M+H-H_2_O] | -- | -- | 819.5892 | 1 | 296.1 | 0.13 | 3 |
| *PG 36:01 | [M+2Na-H] | 288.2 | -0.29 | 821.5328 | 1 | 287.4 | 0.10 | 3 |
| PG 36:01 | [M+2Na-H] | 288.2 | 0.10 | 821.5344 | 2 | 288.5 | 0.13 | 3 |
| PG O-42:05 | [M+H-H_2_O] | -- | -- | 821.6045 | 1 | 300.1 | 0.10 | 3 |
| PG O-44:09 | [M+H-H_2_O] | -- | -- | 841.5738 | 1 | 297.4 | 0.02 | 3 |
| PG O-44:08 | [M+H-H_2_O] | -- | -- | 843.5924 | 1 | 299.5 | 0.14 | 2 |
| **Average % bias** | | | -0.01 | **Average % RSD** | | | 0.09 | |

**Table S3.** Comparison of linear correction factors, average percent biases, and percent relative standard deviations per lipid extract analyzed on the beta prototype (blue portion) and the MOBIE platform (orange portion). The average percent biases and percent relative standard deviations per lipid extract have their respective correction factors applied to the data. Since data was not collected for the PS and SM lipid extracts on the MOBIE platform, an average of the other four lipid subclasses is proposed as the correction factor for PS and SM.
